# The whole versus the sum of some of the parts: toward resolving the apparent controversy of clitoral versus vaginal orgasms

**DOI:** 10.3402/snp.v6.32578

**Published:** 2016-10-25

**Authors:** James G. Pfaus, Gonzalo R. Quintana, Conall Mac Cionnaith, Mayte Parada

**Affiliations:** 1 Department of Psychology, Center for Studies in Behavioral Neurobiology, Concordia University, Montréal, QC, Canada; 2 Department of Psychology, McGill University, Montréal, QC, Canada

**Keywords:** clitoris, vagina, cervix, internal, external, stimulation, arousal, women, pleasure, brain

## Abstract

**Background:**

The nature of a woman’s orgasm has been a source of scientific, political, and cultural debate for over a century. Since the Victorian era, the pendulum has swung from the vagina to the clitoris, and to some extent back again, with the current debate stuck over whether internal sensory structures exist in the vagina that could account for orgasms based largely on their stimulation, or whether stimulation of the external glans clitoris is always necessary for orgasm.

**Method:**

We review the history of the clitoral versus vaginal orgasm debate as it has evolved with conflicting ideas and data from psychiatry and psychoanalysis, epidemiology, evolutionary theory, feminist political theory, physiology, and finally neuroscience.

**Results:**

A new synthesis is presented that acknowledges the enormous potential women have to experience orgasms from one or more sources of sensory input, including the external clitoral glans, internal region around the “G-spot” that corresponds to the internal clitoral bulbs, the cervix, as well as sensory stimulation of non-genital areas such as the nipples.

**Conclusions:**

With experience, stimulation of one or all of these triggering zones are integrated into a “whole” set of sensory inputs, movements, body positions, autonomic arousal, and partner- and contextual-related cues, that reliably induces pleasure and orgasm during masturbation and copulation. The process of integration is iterative and can change across the lifespan with new experiences of orgasm.

Self knowledge is a dangerous thing … The freedom of who you are.

Lou Reed (1989)

You never know where you’re goin’ till you get there.

Jule Styne/Sammy Cahn (1946)

Of all the orgasms on Earth, none are more mysterious than those in females. Controversy has raged over them for more than a century. If they do not serve an obvious reproductive or fitness-related endpoint (Lloyd, 2005; Wallen & Lloyd, 2011; however, see Puts & Dawood, 2006), then why do they exist? What do women get out of them? Can all women have them? And the most mysterious of all: What produces them? This latter question continues to ignite vehement debate over the role of the clitoris and vagina, a debate that we argue stems from at least four sources of misinformation: 1) a misunderstanding of the anatomy and sensory potential of the clitoris, vagina, and cervix (reviewed in Levin, 2015); 2) a misreading of the directionality of sexual differentiation in mammals, in which the clitoris is deemed a ‘vestigial penis’ (e.g. Wilson, 1992); 3) a misattribution of the evolutionary significance of female orgasm, based (as it is in males) on an obvious and direct reproductive function (Symons, 1979); and 4) a misguided assumption that whatever feels good to an individual must also feel good to his (or her) female sex partners. These sources of misinformation stem from definitions of orgasm that essentially reflect a male orgasmic phenotype, male sexual anatomy and physiology, a need for evolutionary necessity, and concepts of normalcy and pathology, all of which are fundamentally flawed when applied to female orgasms. Ultimately, it is a female’s subjective experience, along with her specific anatomy and physiological responses, that defines what is and is not a female orgasm.

## So what is a ‘female orgasm?’

In their classic EPOR (Excitement–Plateau–Orgasm–Resolution) model of human sexual response, Masters and Johnson (1966) described three potential sexual response styles in women, along with three orgasm types that range from one or two of large intensity to multiple orgasms of relatively lower intensity. Some orgasms last a long time, whereas others are of very short duration. Some induce long refractory periods, whereas others seem to come in fairly rapid succession. Of course, the different permutations of intensity, frequency, and location of sensory stimulation give rise to far more than three possible types, depending on the woman’s knowledge of, and comfort with, her own body, her preferred types of sexual stimulation, the degree of automation of sexual motor function (making orgasms easy or difficult to achieve), and the context in which they occur (e.g. masturbation versus partnered, desired and aroused versus routine, hookup versus relationship, and for sexual gratification versus sleep induction).

**Fig. 1:**
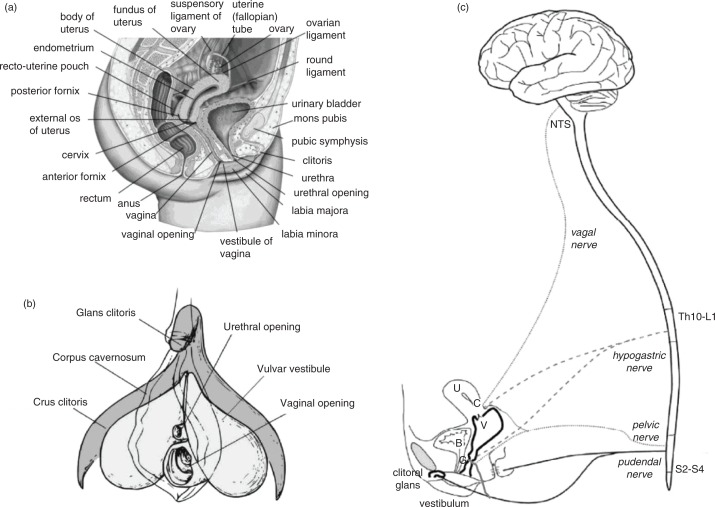
Human female genital anatomy and neurophysiology. (a) Cross-section of the human female genital and pelvic region. (b) The clitoral complex in relation to the urethra, vulva, and vagina. (c) Sensory nerve input to the spinal cord and brain from the genital and pelvic region, including pudendal, pelvic, hypogastric, and vagal nerve innervation. G, G-spot; C, cervix; V, vagina; B, bladder; U, uterus. Adapted from Pfaus et al. (2015) and reprinted with permission.

**Fig. 2:**
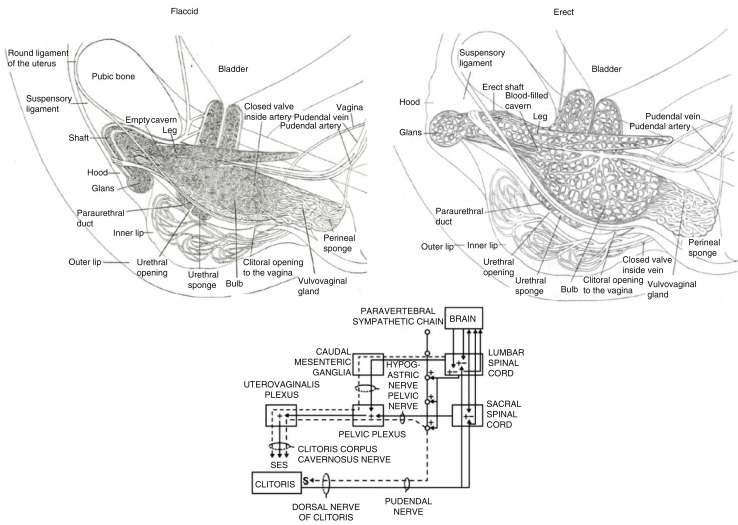
Top: The clitoral complex in its flaccid and erect states. Bottom: Wiring diagram of the sensory and autonomic pathways of the clitoral complex. Adapted from Pfaus et al. (2015) and reprinted with permission.

**Fig. 3:**
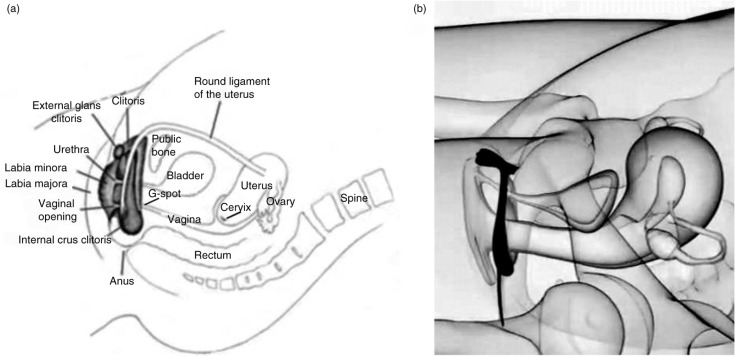
Anatomical placement of the clitoral complex in relation to the vagina, pelvis, and uterus. (a) Drawing depicting anatomical regions. (b) 3-D reconstruction (adapted from Foldes & Buisson, 2009 and reprinted with permission).

**Fig. 4:**
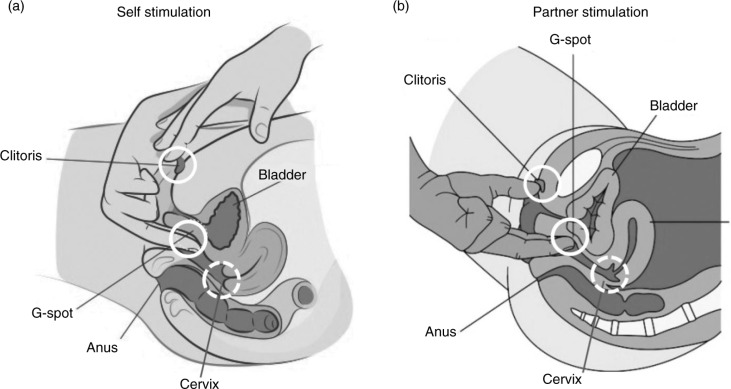
Relative dexterity in stimulating external and internal pelvic structures associated with orgasm from (a) manual self-stimulation, and (b) manual partner stimulation. Adapted from Bodysculptor.com and reprinted with permission.

**Fig. 5:**
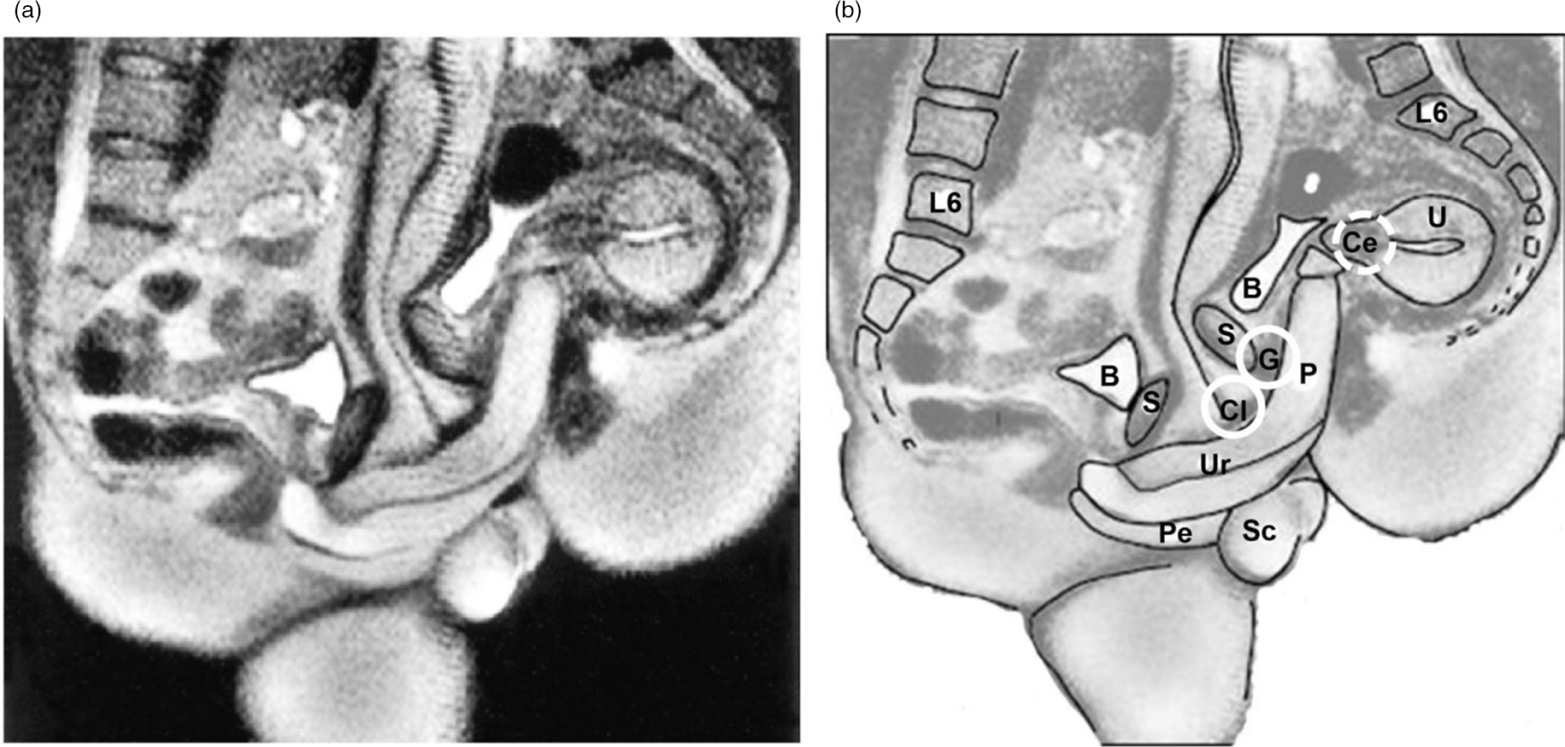
Magnetic resonance image (MRI) of a penis inside a vagina. Participant couple was in the ‘missionary position’. Note the ‘boomerang’ curvature of the penis to meet the shape of the vagina. Left: MRI image. Right: image with approximate anatomical locations of external clitoris, internal clitoris or ‘G-spot,’ and cervix, as in Fig. 4. Images adapted from Schultz, van Andel, Sabelis, and Mooyaart (1999) and reprinted with permission. P, penis; Cl, clitoris; G, G-spot; Ce, cervix; Ur, urethra; Pe, perineum; U, uterus; S, symphysis; B, bladder; Sc, scrotum.

The physiological mechanisms of orgasm have been described for both men and women, and involve nearly identical spinal nerve and brain processes (Fig. 1; see e.g. Komisaruk, Beyer-Flores, & Wipple, 2006; Newman, Reiss, & Northup, 1982; Pfaus, Jones, Flanagan-Cato, & Blaustein, 2015; Salonia et al., 2010; Vance & Wagner, 1976). However, the subjective emotional and cognitive awareness of orgasm, along with its subjective experiences of pleasure, are far less coherent between men and women (e.g. Mah & Binik, 2002, 2005), raising the question of whether orgasms are a singular experience for men and women or whether gender differences truly exist in their expression. This plays into an old controversy concerning the nature of women’s orgasm – where it comes from and what role it plays – in reward or reproduction. For men, orgasm is reached through penile stimulation, the primary end point of which is ejaculation and a subsequent refractory period. Ejaculation is a sympathetically mediated spinal reflex that ejects sperm in seminal fluid, whereas refractoriness is a period of sexual quiescence and inhibition in which the male cannot easily achieve another erection (Hull & Rodríguez-Manzo, 2009, Levin, 2009). Although orgasm in women is not accompanied by seminal emission, some women ‘squirt’ a mixture of urine and Skene’s gland secretions from the urethra at orgasm, which contain urea, creatinine, uric acid, and prostatic-specific antigen (Salama et al., 2015). For women who are able to experience multiple orgasms, the refractory period appears to be shorter between orgasms, but longer after many orgasms have been attained (Salonia et al., 2010). Although for most women orgasm depends critically on stimulation of the external glans of the clitoris, other women are able to achieve ‘different’ kinds of orgasms from stimulation of the external clitoris (Fig. 2), stimulation of select regions inside the vagina, such as the so-called G-spot and/or cervix (Fig. 3), or a blend of the two (Jannini et al., 2012; Komisaruk et al., 2006; Singer & Singer, 1972). Moreover, these can be generated differentially by masturbation or copulation with one or more partners (Figs. 4 and 5). For other women, orgasm is an excruciatingly difficult goal to achieve. Indeed, the inability to ‘let go’ into orgasm, whether by stress or partner-related circumstances, or as a common feature of certain antidepressant medications (e.g. selective serotonin reuptake inhibitors, SSRIs), can become a cause of sexual problems in a relationship (Clayton, Croft, & Handiwala, 2014; Meston, Levin, Sipski, Hull, Heiman, 2004). Although a similar anorgasmia is induced by SSRIs in men (Rowland et al., 2010), endogenous orgasm problems are reported far more frequently by women relative to men (Laumann et al., 2005; Meston et al., 2004).

## Sensory innervation

Four main nerves innervate the female genital tract and related periabdominal regions: the pudendal, hypogastric, pelvic, and vagus, with a relatively moderate degree of anatomical overlap (Figs. 1 and 2). The lower third of the genital tract contains mixed somatic and visceral afferents (with the vulvar vestibule containing a high concentration of somatic afferents). In contrast, the upper two-third is very sparsely innervated with visceral nerves only. These primary afferent nerves produce different qualities of perception that can impact sexual arousal and the quality of genital sensation. Somatic nerves transmit well-localized sensory information with a clear onset/offset and a precise quality, thereby allowing for more immediate peripheral–central feedback. In contrast, visceral nerves convey diffusely localized sensory information with delayed onset in comparison with the stimulation application, and this information may be perceived more slowly unless stimulation is very intense (and intense may or may not be perceived as desirable in this context). The confluence of sharp or punctate fast stimulation relative to diffuse and slow stimulation likely produces differences in regional anatomic sensation that are consciously perceived during sex and that can become associated as primary stimulation for orgasm, as they are for pain (Cervero, 1994).

## Female orgasms, fertility, and reproduction

Clearly ejaculation of sperm is a critical step in sexual reproduction, making the male orgasm easy to conceive of in evolutionary terms (though ejaculation does not always indicate an orgasm, nor is an orgasm in men always indicative of high fertility). What evidence is there that women’s orgasms come with a reproductive benefit? Orgasm induces cervicouterine contractions in women (Shafik, El-Sibai, Shafik, & Shafik, 2005), which has been hypothesized to contribute to an ‘upsuck’ mechanism that facilitates sperm transport, especially if a woman’s orgasm occurs after a male partner has already ejaculated intravaginally (e.g. Baker & Bellis, 1995). Vaginocervical stimulation in a variety of species induces similar uterine contractions and increases sperm transport (England, Moxon, & Freeman, 2012; Michael & Reinke, 1970; Toner & Adler, 1986), an effect likely due to pituitary oxytocin release (Benoussaidh, Maurin, & Rampin, 2005). Orgasm also increases pituitary prolactin release, which should enhance uterine conditions for decidualization and implantation (Krüger et al., 2012). Despite this, orgasm is not a necessary antecedent for fertilization in women (Lloyd, 2005; Singh, Meyer, Zambarano, & Hurlbert, 1998; Wallen & Lloyd, 2011), and the potential reproductive benefits of a cervicouterine ‘upsuck’ mechanism in humans remain hypothetical. Although orgasms may well facilitate mate-choice, honing the neurochemical mechanisms of attention and bonding to salient partner-related cues associated with sexual pleasure (Pfaus et al., 2012; Puts, Dawood, & Welling, 2012), that is hardly a direct effect on reproduction or fertility. Of course, one can ask whether female orgasms *must* enhance reproduction directly to be deemed important from physiological, psychological, or even evolutionary standpoints. Clearly, they are not some vestigial version of a male orgasm, because functional sexual differentiation of the gonads, genitals (and brain) in mammals moves from a bipotential precursor to male phenotype as a result of androgen and estrogen actions, with the ‘default’ effect of no steroid hormone action being the development of the female phenotype (Nelson, 2011; Wilhelm, Palmer, & Koopman, 2007; however, see McCarthy & Arnold, 2011). It is, therefore, impossible that anything in female mammals represents a ‘vestigial’ male phenotype or function.

## Variability of experience: the role of the clitoris versus the vagina

Another problem eluded to above concerns the variability in the experience of orgasms in women. Some women have them regularly whereas others do not. Some never have them. Some can only have them through masturbation, whereas others differentiate the pleasure they receive from the blending of clitoral and vaginal sensations with a partner relative to manual stimulation of the external clitoris alone (e.g. Buisson & Jannini, 2013). This variability exists despite the plethora of self-help sex manuals for women. What does this variability reflect? Has it always been this way? Can’t all women experience orgasm?

It seems that for thousands of years, including most of the first 2,000 years of the common era (CE), pleasure from sex in general, and orgasms in particular, were assumed for both women and men across many different cultures. In ancient Greek mythology, Tiresias the Seer was made blind by Hera for agreeing with Zeus that women experience greater pleasure than men from sex. Ancient sexual texts like the *Kama Sutra of Vātsyāyana* (3rd Century CE; 1925) regarded the pleasure from sex that women and men experience as natural and potentially equal, even if the physical means of obtaining it were different. The Taoist *Art of the Bedchamber* books (Wile, 1992) emphasized that women needed to be stimulated properly to achieve pleasure and orgasm from sex, and that women’s sexual abilities and pleasures were their own, and did not exist simply to please men. In Japan, *Shunga* art up to the end of the 19th Century depicted women in various states of sexual ecstasy from a variety of heterosexual, homosexual, and fetish sex practices (Buckland, 2013). Similarly, European erotic art from the 17th to the early 19th Centuries depicted women as taking an equal part in different types of sex play and with facial expressions denoting extreme pleasure and orgasm (e.g. the famous painting of *Cupid and Psyche* by Jacques Louis David (1817); found in Neret, 2001).

The role of genital stimulation in producing sexual pleasure was less obvious. Chalker (2000) describes how the clitoris was viewed as a tiny phallus for thousands of years, equal to the penis in terms of the generation of pleasure. However, certain outspoken physicians like Galen in the 2nd Century, and later Vesalius in the 16th Century, viewed the vagina as an ‘inverted penis’ more important for women’s reproductive (and therefore sexual) pleasure. A number of anatomists, notably Estienne in 1547, Columboin 1559, and Falloppio in 1561, laid claim to having ‘discovered’ (meaning rediscovered) the clitoris and provided detailed anatomical drawings from cadavers that included its nervous connections. Debate about its role in sex and reproduction came with the work of Bartholin and de Graaf in the 17th Century and Kobelt in the 19th Century (reviewed in O’Connell, Sanjeevan, & Hutson, 2005). The notion of the vagina as the main female sex ‘organ’ took hold in medical texts of the 19th Century, with the clitoris all but forgotten. Interestingly, since the time of Hippocrates, a mix of clitoral and vaginal stimulation to orgasm had been used to ‘treat’ so-called hysteria (Maines, 1999). Although 19th Century Victorian-era doctors did not believe that women experienced sexual desire or anything approximating the pleasure of a ‘male’ orgasm during sex (since neither were viewed as a ‘requirement’ for reproduction in women), they often prescribed the age-old, doctor-assisted genital stimulation that induced ‘paroxysms’ to treat hysteria. Such stimulation generally consisted of internal vaginal stimulation with a straight vibrator, often in combination with manual massage of the external clitoris with the fingers or a water spray. The ensuing paroxysm was hypothesized to center the wandering uterus back into its ‘normal’ position.

Could women be taught to do this themselves? Evidently not, at least not openly. The latter half of the 19th Century also saw the advent of organized religious attacks on masturbation (‘Onanism’ described as ‘self-abuse’ or ‘self-pollution’) in the West. Masturbation was believed to cause all manner of physical, mental, and ‘moral’ disorders in adults (Katsainos, 1929) and lead to malformed brains (Vorhees & Hannaford, 2014). Stipulations against it were many, appearing in both medical and popular texts (e.g. Fowler, 1870), and offenses were punished harshly (Waxman, 2007). Despite this, many women secretly used ‘muscle beaters’ with handles to massage their genitals for self-gratification. Indeed, electric vibrators were sold by mail order (e.g. from successive catalogs of Sears, Roebuck and Co.) up to the start of World War II as ‘Aides that Every Woman Appreciates’. Although these seemed to disappear in the 1940s and 1950s, they returned in the 1960s and remain to the present day specifically as ‘sex toys’ marketed largely to women (Maines, 1999).

## Freud’s dichotomy

Resistance to the Victorian era’s attempts to muzzle free sexual expression and regulate human reproduction came from a few sources, notably physicians in the new clinical domain of psychoanalysis spearheaded by Sigmund Freud. The psychoanalytic movement believed that social repression of sexuality led to neuroses (Guyon, 1934; Reich, 1942/1973) and used the analysis of symbolic cognitive content from dreams, free associations, fantasies, Rorschach ink-blots, and so on, to derive an idea of where a neurotic individual was stuck (Jastrow, 1948). In his *Three Essays on the Theory of Sexuality*, Freud (1905/1962) outlined putative stages of sexual development through childhood and adolescence. He distinguished two phases of genital awareness during development, one called the ‘phallic-oedipal stage’ (ages 3–6) and one called the ‘mature genital stage’ (puberty onward). The former stage reflected genital awareness for its own sake, in which pre-erotic pleasure is derived from touching the genitals. The latter stage reflected genital awareness in a physically mature body, upon which genital stimulation serves the larger goals of sexual pleasure and reproduction. The ‘infantile’ body was, in Freud’s terminology, ‘polymorphously perverse’, meaning that all areas of the body could experience pleasure in their own right. In contrast, the ‘mature’ body (and brain) parsed pleasurable sensations out to specific zones and for specific purposes. For example, to a child, all areas of the body can be tickled, and tickling is fun in its own right. Children lose this capacity during puberty; as the ticklish child becomes a reproductively mature adult, tickling can be fun or torture depending on the context.

Unfortunately, in this theory Freud declared that clitoral stimulation and the orgasms derived from it represented an ‘infantile’ state of pleasure, not unlike the pleasure derived from brief episodes of penile stimulation in little boys. The orgasms produced by clitoral stimulation were considered superficial and entirely genitally based. ‘Mature’ orgasms, on the other hand, were derived exclusively from vaginal stimulation, not unlike the orgasms of adult males who stimulate the penis in order to ejaculate. These orgasms were ‘deeper’ and felt throughout the entire body. In essence, clitoral pleasure was supposed to ‘transfer’ to the vagina during puberty. These ideas rested on no empirical evidence whatsoever, although it was common at that time to believe that hysteria required vaginal stimulation to produce the paroxysms necessary as a ‘cure’ and that deep vaginal orgasms were thought to enhance reproduction, unlike clitoral orgasms. Thus, a woman requiring clitoral stimulation to achieve orgasm was believed to be ‘stuck’ in a more infantile phallic stage of her psychosexual development. One goal of psychoanalysis for such a woman was to overcome neuroses, in part, by learning how to transfer clitoral pleasure into full vaginal pleasure.

## Round 1: the return of the clitoris

The qualitative judgmental division between clitoral (‘infantile’) and vaginal (‘mature’) orgasms found its way from psychoanalytic interpretation to psychological, psychiatric, and medical practice to everyday life. For the first half of the 20th Century, it was simply assumed that vaginal orgasms were the only ‘real’ orgasms (if, indeed, orgasm occurred at all in women) and that women who experienced orgasm only from direct stimulation of the clitoris were considered stuck in an egotistic, child-like state of sexuality (e.g. Willy, Vander, & Fisher, 1950). Despite this, clitoral stimulation by means of vulvar friction, stimulation of the clitoral sheath, and/or direct stimulation of the clitoral glans and labia was the most common form of masturbation by women in many different cultures (Ford & Beach, 1951). Kinsey, Pomery, Martin, and Gebhard (1953) reported this for their North American female sample, in addition to the admission by many women that secret stimulation of the clitoris during heterosexual intercourse was the only way they could actually achieve orgasm. Kinsey et al. referred to the vagina as an ‘insensitive orifice’ with relative importance for reproduction, but relative unimportance for erotic gratification. It was poorly innervated neurologically relative to the clitoris, thus supporting the latter as the main source of sexual pleasure in women. With regard to orgasms induced by penile–vaginal stimulation alone, Kinsey et al. (1953) noted: ‘Some hundreds of the women in our own study and many thousands of the patients of certain clinicians have consequently been much disturbed by their failure to accomplish this biologic impossibility’ (p. 584).

Subsequently, a plethora of evidence came into view supporting the external clitoris as ‘THE’ female sexual organ of pleasure and orgasm. Masters and Johnson (1966) reported that most women achieved orgasm from clitoral stimulation, whereas far fewer achieved it from vaginal stimulation. They noted that the clitoral structures (e.g. corpus cavernosum and bulb of the vestibule) extend below the labia, making it possible for penile intromissions in the right position to stimulate the clitoris indirectly by moving these structures upward. Although Shere Hite (1976) criticized Masters and Johnson for their use of a biased sample of women who could experience orgasm during penile–vaginal intercourse, her report on female sexuality essentially replicated Masters and Johnson’s and Kinsey’s findings. Although some women (approximately 26%) had the ability to experience vaginal orgasm without accompanying clitoral stimulation, most (approximately 70%) experienced orgasms exclusively from clitoral stimulation.

During this time, the clitoris also became a sociopolitical flag-bearer. Many feminist scholars and collectives denounced the Freudian view of vaginal orgasm (e.g. Boston Women’s Collective, 1970; Koedt, 1970). Anne Koedt was one of the most vocal. She was adamant that women who claim to have vaginal orgasms are either confused about the locus of sensations (believing that they originate in the vagina during intromissive penile stimulation) or are actively deceiving men by ‘faking it to get ahead’. Maintaining the myth of vaginal orgasm was viewed as maintaining male perceptions of the vagina as an ultimate source of stimulation for the penis and fed the notion that female pleasure is subsumed by male pleasure (what feels good to him must also feel good to her). The blatant disregard for the clitoris in the modern era was seen as an example of the patriarchy fearing female sexuality in general, and the clitoris in particular. Recognition of clitoral orgasm would threaten the entire notion of heterosexuality as an institution and the role of the penis as a provider of pleasure to women. Advancing this theme further, Andrea Dworkin (1987) argued that penetration was subjugation of women by men: ‘The vagina itself is muscled and the muscles have to be pushed apart. The thrusting is persistent invasion. She is opened up, split down the center. She is occupied – physically, internally, in her privacy … There is no analogue anywhere among subordinated groups of people to this experience of being made for intercourse: for penetration, entry, occupation’ (pp. 122–124). So it was the clitoris all along! The patriarchy had tried to demonize it, but thanks to Kinsey, Masters and Johnson, and Hite, and the millions of women in the West who risked neuroses to achieve sexual gratification, they failed. Women’s subjective experiences of what caused orgasms had to be considered rather than discounted. Clitoris 1: Vagina 0.

## Round 2: the vagina strikes back

During the early 1980s, a number of popular men’s magazines started raging about ‘female ejaculation’. The German gynecologist Ernst Gräfenberg, known for (re)inventing the intrauterine device in the 1920s, reported cases of women who discharged an ejaculate-like substance from the urethra during orgasms induced by stimulation of the anterior vaginal wall (Gräfenberg, 1950). Cases of female ejaculation had been reported previously by Krafft-Ebing (1886/1924) and Freud (1905/1962) and were also found in anthropological works, notably in Malinowski’s (1928) study of the Trobriand Island people, along with women in many other primitive cultures (e.g. Gladwin & Sarason, 1956). Following a series of case histories and a review (Addiego et al., 1981; Beltzer, 1981; Perry & Whipple, 1981; Sevely & Bennett, 1978), Ladas, Whipple, and Perry (1982) published their popular book on the so-called G-spot (named after Gräffenberg). The spot was hypothesized as a ‘female prostate’ that should be found in the anterior wall between the opening of the vagina and the urethra. However, this region also contains the periurethral glands known as ‘Skene’s glands’ that secrete fluid. Upward pressure on the upper curved wall of the vagina puts pressure on these glands which can result in secretion of fluid that resembles semen. In addition, more recent ultrasounds have shown that the bladder fills during sexual intercourse and contracts in women who ‘squirt’ fluid from the urethra at orgasm (Salama et al., 2015). Whether the secretion is from Skene’s gland or bladder, or whether it occurred at all, manual or vibratory stimulation of the G-spot alone was reported to induce a deep orgasm in almost half of a large sample of professional women that responded to an orgasm questionnaire (Darling, Davidson, & Conway-Welsh, 1990).

Could women truly distinguish between orgasms induced by clitoral or vaginal stimulation? Butler (1976) and Clifford (1978) reported that sexually active undergraduate women could indeed distinguish clitoral from vaginal orgasms. Through self-repot, these women described a clitoral orgasm as localized, intense and physically satisfying, whereas a vaginal orgasm was described as stronger and longer lasting than clitoral orgasm, ‘deeper’, a ‘whole body’ sensation with throbbing feelings, and more psychologically satisfying. However, despite these subjective differences, but there was no clear subjective winner between the two, and most women utilized some blended stimulation of the two to achieve orgasm. Leff and Israel (1983) found that women who masturbated via clitoral stimulation alone had a significant preference for clitoral orgasms and were significantly more likely to use clitoral stimulation during penile–vaginal intercourse to stimulate themselves to orgasm. Women classified as non-masturbators achieved orgasm less frequently overall and did not typically use clitoral stimulation to do so. Interestingly, a study by Chambless et al. (1982) examined the relationship of pelvic floor (pubococcygeal) muscle strength and orgasm. Pubococcygeal strength was not related to frequency or self-reported intensity of orgasm, nor did it predict the occurrence of vaginal orgasm. However, it was correlated with the pleasure achieved using clitoral stimulation to orgasm, suggesting that pelvic floor contractions at orgasm can be induced by clitoral stimulation alone. A study by Sholty et al. (1984) reported that most women experienced orgasm during intercourse and that the experience was based in the clitoral/vaginal region. However, women over 40 years of age were more likely to have experienced orgasm by stimulation of more than one region of the body, whereas women between the ages of 18 and 29 were more likely to have experienced orgasm by clitoral stimulation alone. Thus, one’s own experience of orgasm seemed to reinforce the physical manner in which it was obtained, and there seemed to be a connection between clitoral stimulation and vaginal/uterine responses during orgasm (echoing the notion by Koedt that women could stimulate the clitoris but ‘feel’ the orgasm elsewhere; Singer & Singer, 1972).

The existence of the G-spot received much press and widespread acceptance among women who differentiated clitoral from vaginal orgasms. However, it was also met with skepticism by clinicians (e.g. Hines, 2001) and researchers (e.g. Levin, 2003) and remains controversial specifically because of numerous failures to determine its existence as a ‘female prostate’, much less the ability of all women to find it, stimulate it, and have it induce orgasm (e.g. Kilchevsky, Vardi, Lowenstein, and Gruenwald, 2012; Pan, Leung, Shah, & Kilchevsky, 2015). A twin study by Burri, Cherkas, and Spector (2010) was one of the most glaring. Of 1,804 female twins aged 22–83 who completed a questionnaire that included questions about the presence or absence of a G-spot, only 56% reported having a discernable G-spot, and the prevalence decreased with age (rather than increased, as might have been expected from the results of Sholty et al. 1984). Variation in the frequency of reported G-spots was almost entirely a result of individual experience and random measurement error (>89%), with no detectable genetic influence. So what was this fleeting anatomical anomaly that some women swore by and others swore at?

A series of anatomical studies using histology and MRI by O’Connell and colleagues (O’Connell, Eizenberg, Rahman, & Cleeve, 2008; O’Connell, et al., 2005; O’Connell, Hutson, Anderson, & Plenter, 1998) revealed that the anatomical location of the G-spot was contiguous with the inner legs or roots of the clitoris and the erectile tissue of the clitoral bulbs and corpora, along with the urethra, and argued that these form a coherent anatomical ‘whole’ that share common vasculature, nerve supply, and responses during sexual stimulation. Indeed, the anterior wall of the vagina was found to have more nerve endings compared to the posterior wall and found in sub-epithelial layers (Hilleges, Falconer, Ekman-Ordeberg, and Johansson, 1995; Song, Hwang, Kim, & Han, 2009). These sensory nerves course into the dorsal clitoral nerve, connecting with the sensory nerves coming from the external glans of the clitoris and entering the pudendal nerve plexus. Foldes and Buisson (2009) came to the same conclusion using 3-D sonography of the stimulated clitoris, showing that, when engorged, the internal portions of the clitoris surround the anterior vaginal wall (Figs. 2 and 3). They referred to this as ‘the clitoral complex’ and denoted both external and internal parts. The engorged clitoris, then, increased the likelihood that orgasm could be experienced from both external clitoral glans stimulation and internal stimulation of the clitoral root (posterior portions of the clitoral complex) that folded around the anterior vaginal wall. Buisson, Foldes, Jannini, and Mimoun (2010) evaluated the interaction between the vagina and clitoris during coitus, visualizing live through an ultrasound scanner the anterior vaginal wall and its relation to the clitoris during penile–vaginal intercourse. The volunteer woman was on her back with her legs up when her male partner penetrated her. Results demonstrated that the penis inflated the vagina, stretched and widened the root of the clitoris. During penile thrusting, the anterior wall of the vagina crashed with each thrust against the root of the clitoris, a possible explanation of the sensitivity claimed by women during penetration, thus revealing a very real relationship between these structures during intromissive vaginal penetration. Thus, the root of the clitoris and the anterior wall of the vagina appear to be anatomically and functionally related, providing an explanation why some women can achieve orgasm by vaginal stimulation alone. Vehement opposition to those findings was expressed by Vincenzo Puppo (2011a, 2011b), who felt that the clitoral bulbs are more properly called the vestibular bulbs and do not belong to the clitoris. Thus, there is no such thing as an internal clitoris that a penis or phallic sex toy should ever be able to come into contact with, especially given the 10–12 mm thickness of the vaginal wall (albeit in an unaroused state). And, if it did exist, then why can’t all women experience orgasm from internal stimulation? However, although others agree that the clitoral roots could be stimulated internally to produce orgasm, they note concerns about the location of the G-spot as a distinct anatomic sensory entity (e.g. Kilchevsky et al., 2012).

But another region inside the vagina also emerged as a source of sensory stimulation related to orgasm. Stimulation of the cervix had been known in animals to abbreviate the period of behavioral estrus and induce analgesia (Crowley, Jacobs, Volpe, Rodriguez-Sierra & Komisaruk, 1976; Komisaruk & Steinman, 1986; Lodder & Zeilmaker, 1976; Pfaus, Smith, Byrne, & Stephens, 2000). In a series of papers, Komisaruk and colleagues found that direct cervical stimulation could induce orgasm in women with complete spinal transection at T10 or higher, implicating the cervix and a hitherto unknown neuroanatomic linkage to the vagus nerve in transmitting pleasurable orgasmic sensations (Komisaruk & Whipple, 2005; Komisaruk et al., 2004, 2006; Whipple & Komisaruk, 2002). Pelvic floor contractions during orgasm produce cervicouterine contractions (Shafik, 1995) that may form part of the ‘upsuck’ mechanism that is hypothesized to aid sperm transport to the uterus. Cervical stimulation may also be critical for increased prolactin secretion from the anterior pituitary that maintains progesterone secretion from the corpora lutea that is critical for fetal implantation and growth prior to full placental formation (Pfaus et al., 2015). Thus, in addition to its potential role in reproduction, cervical stimulation itself may be a second intravaginal trigger of orgasm after the internal clitoris.

So which is it? Clitoral or vaginal? Both? And which sensory region of the vagina? Does one win at the expense of the other? And what about women who experience orgasms from intense nipple, neck, and/or outer and inner ear stimulation? What about women who can work themselves up to orgasm from imagery alone (e.g. Whipple, Ogden, & Komisaruk, 1992)?

## A new hope: the personal orgasm dialectic

The current state of the debate over women’s orgasm continues to posit the clitoris against the vagina and is punctuated on both sides by the writings of two men: Vincenzo Puppo arguing for the clitoris and Stuart Brody arguing for the vagina. Puppo (2011a, 2011b), Puppo and Gruenwald (2012), Puppo and Puppo (2014) argue that women’s orgasm cannot be vaginal because there is nothing in the vagina – no stimulation of an internal clitoral complex and no stimulation of the cervix – that could possibly stimulate orgasm. Orgasms are generated by stimulation of the external clitoral glans alone, and women should not suffer the frustration of trying to obtain orgasm from vaginal stimulation and the indignities of self-doubt when those orgasms do not occur. In contrast, Brody and colleagues (e.g. Brody, 2010; Brody & Costa, 2013; Brody & Weiss, 2010, Brody & Weiss, 2011; Costa & Brody, 2012) argue that women’s sexual satisfaction and general psychological well-being depend on their specific experience of vaginal orgasm. Brody (2012) even argues that the consistency with which women experience vaginal orgasm is a physiological ‘trait’ in those women, one that is negatively related to the discordance other women experience between genital and subjective measures of sexual arousal (e.g. Chivers, Seto, Lalumière, Laan, and Grimbos, 2010). Interestingly, Brody, Klapilová, and Krejčová (2013) suggested that experiencing vaginal orgasms increases the sensitivity of the vagina to intromissive stimulation, and Leeners et al. (2013) reported that the more pleasurable the orgasm from penile–vaginal intercourse, the greater the release of prolactin from the pituitary. Of course, clitoral stimulation alone, and the use of clitoral stimulation during penile–vaginal intercourse, was not examined in that latter study.

**Fig. 6:**
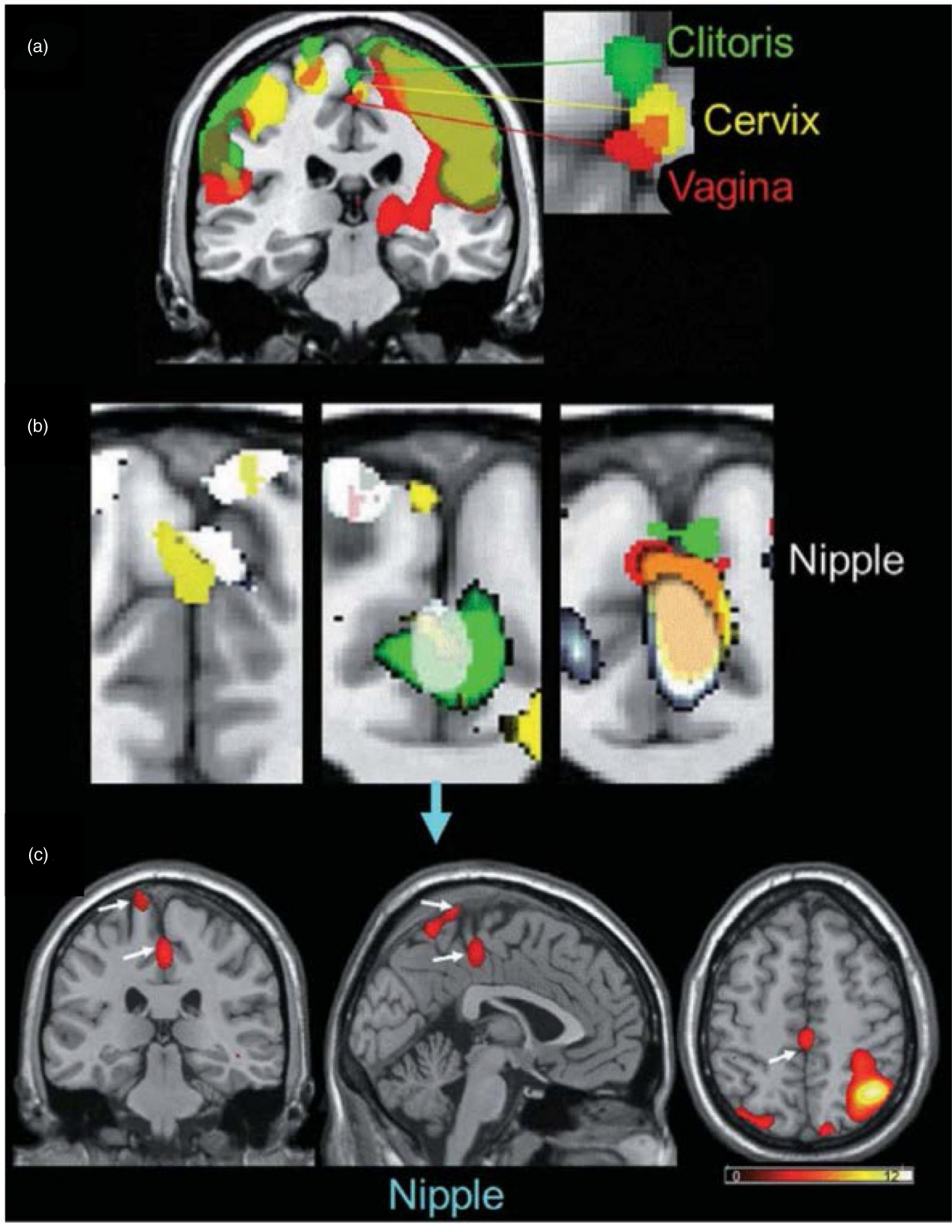
Brain activation patterns derived from fMRI scans during stimulation of the external clitoris, vagina, cervix, and nipples. From Komisaruk et al. (2011). Reprinted with permission.

So how can this be resolved? One way is to acknowledge that they may both be right to some extent. It is likely that women have an *enormous* capacity to experience orgasms of many different types (e.g. as suggested by Jannini et al., 2012). Although orgasm is a spinal reflex (Komisaruk et al., 2006), the subjective experience of it is not necessarily the same for each woman, and can even be different each time a woman has one. Those differences span physiology and psychology. They depend on the unique distribution of sensory nerves in the external glans of the clitoris, the internal distribution of sensory nerves around the posterior clitoral complex and cervix, the integrity of the nerves that transmit those sensations into the spinal cord and/or directly to brain, and, perhaps most importantly, a woman’s relative experience with sensory stimulation of one, some, or all of those regions being associated with orgasm. Indeed, using fMRI, Komisaruk et al. (2011) reported that specific but partially overlapping zones of somatosensory cortex are activated by specific stimulation of the external clitoris, anterior vagina, cervix, and nipples (Fig. 6). The partial overlap of these somatosensory processing zones raises the possibility of both bottom-up and top-down sensory blending of the stimulation that induces orgasm and the region(s) of the body that the orgasm(s) are experienced in. As noted, brain activation depends on the ‘hardware’ of nerves (and their interconnections that link processing units of the brainstem, midbrain, hypothalamus, thalamus, limbic system, and cortex) and on the genetic and epigenetic ‘software’ that underlies experience-dependent behavioral plasticity (Sweatt, 2016). It is likely, therefore, that the type of stimulation provided by a partner (not only genital, but of every sensory modality, including olfactory and potentially pheromonal), the preferred method(s) of self-stimulation used during sex, and the particular sexual positions used, become predictive of sexual arousal and ease (or difficulty) of orgasm. That some swear by one and against others is simply a reflection of this diversity of experience.

Perhaps it is time to stop treating women’s orgasm as a sociopolitical entity with different sides telling women what they can and cannot experience or debating whether female orgasm is a vestigial male orgasm. As mentioned above, mammalian males differentiate from what would have become a female ‘default’ phenotype, not the other way around (Nelson, 2011). It is time to stop making believe that the vagina is an inverted penis or that the clitoris is somehow a ‘vestigial penis’ with a smaller capacity for stimulation and pleasure relative to the penis. And to the chagrin of an unfortunate number of men, it is time to stop acting like sexual interaction begins and ends with an erect penis. Sexual gratification in women *never required a penis or penis-shaped sex toy*, though having one that is responsive and attached to someone who is interested in exploring and cultivating her sexual landscape, can embellish her experience and augment the array of sensory stimulation that she can associate with orgasm.

## Role of learning

Association with orgasm is a key to understanding the role played by the clitoris and internal vaginal structures, as well as motor movements during masturbation and copulation that become a crystallized pattern. The entire experience of sexual interaction to orgasm can be viewed from the perspectives of both Pavlovian conditioning and operant responding. One’s first experience of orgasm is likely the product of masturbation. The movements around the external clitoris, just like movements up and down the penile shaft, become operants that lead more and more reliably to orgasm. These movements become crystallized and automated with experience, allowing the masturbator more cognitive leeway to fantasize and toggle through different types of erotic visual and auditory stimuli. Although such stimuli are typically prepotent in terms of inducing arousal early on in the experience, orgasm as a state of pleasure can be viewed as a Pavlovian unconditioned stimulus (UCS) that is typically predicted by preferred types of erotic cues, which themselves are conditioned stimuli (CSs), along with preferred operant somatosensory stimulation that engages the sympathetic outflow necessary for orgasm. Mastery over this process is part of the sexual self-knowledge, discovery, and confidence that every person has a right to experience and should have coming into – and enjoying – sexual interaction with another.

First sexual experiences with a partner engage new sensory domains. New faces, new bodies, new grimaces, new smells, new sounds, new fingers and tongues, and new styles of stimulation (penetration, thrusting, and caressing) can all conspire to increase (or decrease) overall arousal and pleasure. An erect penis or sex toy inside the vagina can stimulate the internal clitoris and perhaps even the cervix itself depending on the sexual position used and the particular anatomy of the woman’s vagina, cervix, and uterus (Figs. 4 and 5). The penis or toy also provides intravaginal pressure that most women are aware of. With sensory stimulation of the external clitoris as orgasm approaches, a woman may well achieve a ‘new’ type or quality of orgasm. And once achieved, her brain will automatically differentiate the two and place them into a conscious, cognitive, emotional, visceral, and often linear, perspective. Once automated with experience, and with sufficient sympathetic arousal induced by fantasy or real external cuing, it is possible that ‘penile stimulation alone’ (of the internal clitoris and/or cervix) can induce orgasm. This likely depends on several variables, including the sexual position(s) used, which in the case of heterosexual intercourse or the use of a phallic sex toy can dramatically alter the position of internal organs and differentially stimulate pleasurable sensory zones (Faix, Lapray, Callede, Maubon, & Lanfrey, 2002), and the sensory thresholds of these three regions, which are likely defined individually. In fact, the group differences in orgasm consistency observed in the studies of Brody and colleagues may well be indicative of a cluster of women who possess low sensory thresholds within the two vaginal sensory regions. Obviously a woman who never goes there, or who has never received competent stimulation with a toy or by a partner, will simply not know – *until she does*. And her knowledge of this likely changes across the lifespan, as she allows herself to experience different types of sexual stimulation in different contexts and/or with different partners. This would be expected to result, for example, in the honing of assessments of ‘peak’ or optimal sexual experiences as people age (e.g. Kleinplatz & Ménard, 2007). It is not yet known whether one or more sensitive periods might exist in the development of orgasm ability, or whether different types of orgasms experienced at different times in the lifespan result in a modification of the central neurochemical systems activated before or during orgasm, such as dopamine, oxytocin, vasopressin, opioids, serotonin, and endocannabinoids (Pfaus, 2009).

Motor movements too become conditioned patterns that predict orgasm. Not only the movements of fingers and hands around the external clitoris, but trunk and hip movements (which are typically constrained by the sex positions used), along with facial, jaw, and neck tension held as orgasm approaches (not to mention heart rate, breathing rate, and repetitive moans or emotional utterances), can be activators of sympathetic and central arousal, and thus necessary antecedents of orgasm. Sensations of muscle movement inside the vagina and around the pelvic floor if a penis or toy is inserted, along with the movements of abdominal viscera, may also become associated with orgasms in general or differentiated among different types. Indeed, it is possible that some of the regions of confluence in the somatosensory cortex shown in Fig. 6 by Komisaruk et al. (2011) reflect spillover activation of the adjacent precentral gyrus, thus reflecting movement rather than location of the sensory stimulation. Moreover, awareness of pleasurable orgasmic sensations centered in the genitals are likely differentiated from more diffuse sensations in the entire hypogastric plexus, but are encoded along with the movements (or fantasy-laden representations of the movements) that create them. Inspired use of the adjectival Orgasm Rating Scale by Mah and Binik (2002) could begin to make sense of how different types of orgasm coming from different types of stimulation might be interpreted.

## Lessons from lab rats

In sexually naïve female rats, distributed stimulation of the external clitoris with a fine paintbrush – applied in a manner that simulates the clitoral stimulation she would receive during multiple bouts of paced copulation with a male – generates its own reward state and activates regions of the brain associated with sexual desire and pleasure (Parada, Chamas, Censi, Coria-Avila, & Pfaus, 2010). However, when females have prior paced sexual experience with a male, which also induces a sexual reward state (Paredes & Vazquez, 1999), external clitoral stimulation alone, although clearly pleasurable, does not generate a full reward state but instead induces females to make more sexual solicitations of males (Parada, Jafari, & Pfaus, 2013). In short, what was a full meal to the naïve female becomes an hors d’oeuvre to the experienced female. Could this result from a differentiation of copulatory stimulation as full (external and internal) clitoral stimulation occurring along with cervical stimulation, flank stimulation, and olfactory/pheromonal stimulation, relative to stimulation of the external clitoris alone? Could something analogous happen to a woman with a long history of external clitoral stimulation to orgasm but who experiences for the first time a ‘different’ orgasm from internal clitoral and/or cervical stimulation (not to mention her first orgasm produced by nipple stimulation or stimulation of the cymba conchae of the outer ear; see Komisaruk et al., 2011; Frangos, Ellrich, & Komisaruk, 2015)?

Although paced copulation is necessary to induce a reward state in female rats, the type of clitoral and vaginal stimulation a female receives differs if the male is more or less vigorous. In turn, this makes a difference for the induction of a progestational state, in addition to the development of conditioned place and partner preferences. To induce a progestational state, female rats require more – and more vigorous – intromissions (e.g. Adler, 1969, 1978; Erskine, Kornberg, & Cherry, 1989). The pelvis of the mounting male makes contact with the external clitoris during mounts with pelvic thrusting (Pfaff, Montgomery, & Lewis, 1977). If the male’s penis is erect during these thrusts then vaginocervical stimulation will also occur with each successive intromission and occur for a longer duration during ejaculation and the deposition of a gelatinous sperm plug. Male rats copulating with females in a unilevel pacing chamber make more vigorous mounts and display a greater number of intromissions prior to ejaculation if the divider between the two sides has a single hole for the female to cross back and forth, relative to one with four holes (Ismail, Zhao, & Pfaus, 2008). Although both males and females show conditioned place preference for the four-hole condition relative to the one-hole condition, females develop a conditioned partner preference and mate guarding of a male only if they are paired with the same male in the one-hole condition. It is possible that greater arousal before and/or during copulation leads to a more powerful reward state, one that links the activation of oxytocin, vasopressin, dopamine, and even gonadotropin releasing hormone systems, to partner-related cues.

## Conclusions

The distinction between different orgasms, then, is not between sensations of the external clitoris and internal vagina, but between levels of what a woman understands a ‘whole’ orgasm to consist of. This depends on the experience with direct stimulation of the external clitoris, internal clitoris, and/or cervix, but also with knowledge of the arousing and erotic cues that predict orgasm, knowledge of her own pattern of movements that lead to it, and experience with stimulation of multiple external and internal genital and extra-genital sites (e.g. lips, nipples, ears, neck, fingers, and toes) that can be associated with it. Orgasms do not have to come from one site, nor from all sites; and they do not have to be the same for every woman, nor for every sexual experience even in the same woman, to be *whole* and *valid*. And it is likely that such knowledge changes across the lifespan, as women experience different kinds of orgasms from different types of sensations in different contexts and/or with different partners. Thus, what constitutes a ‘whole’ orgasm depends on how a woman sums the parts and the individual manner in which she scales them along flexible dimensions of arousal, desire, and pleasure. The erotic body map a woman possesses is not etched in stone, but rather is an ongoing process of experience, discovery, and construction which depends on her brain’s ability to create optimality between the habits of what she expects and an openness to new experiences. Most importantly, the application of a restricted reproductive model of male ejaculation to understanding the cause and effect of women’s orgasms only serves to obfuscate and hide the truly remarkable variety of orgasmic experiences a woman can have.
